# Retinopathy of prematurity in Bangladesh: an overview

**Published:** 2017-11-11

**Authors:** Nazmun Nahar, Sarat Adolore Badmus, Sanjoy Kumer Das, Mohammad Ibn Abdul Malek, Mostafizur Rahman, Mohammad Abdul Mahid Khan

**Affiliations:** 1 Associate Professor: Ispahani Islamia Eye Institute and Hospital; 2 Fellow Paediatric Ophthalmology: Ispahani Islamia Eye Institute and Hospital; 3 Consultant: Ispahani Islamia Eye Institute and Hospital.; 4 Consultant: Ispahani Islamia Eye Institute and Hospital.; 5 Senior Consultant: Ispahani Islamia Eye Institute and Hospital.; 6 Senior Consultant: Ispahani Islamia Eye Institute and Hospital.

**National ROP guidelines, database for monitoring, evidence-based policy making, and provision of infrastructure and equipment are critical to prevent a ROP epidemic in Bangladesh.**

Bangladesh is the eighth most populous country in the world and currently a lower middle income country.^1^The improvement in the country's economy has led to a visible improvement in its neonatal care. As more premature babies now survive, the incidence of retinopathy of prematurity (ROP) is also on the rise. This improvement in neonatal care, which is sometimes suboptimal because of limited facilities and manpower contributes to the current epidemic of ROP in Bangladesh.

Approximately 3.75 million infants are born in Bangladesh each year. About 25,000 of these weigh 1,500 grams or less (and hence at risk for ROP).^2^ From our experience, even bigger babies may be at risk because of inadequate monitoring of oxygen and difficulty with management of perinatal complications.

**Figure:**
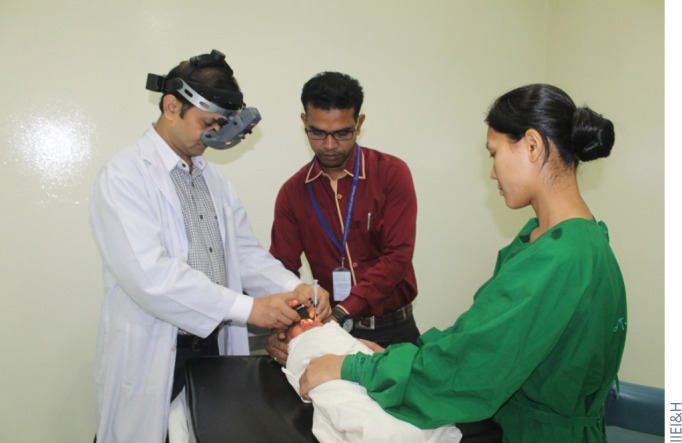
ROP screening at IIEIH. BANGLADESH

One of the earlier studies for ROP in Bangladesh included preterm infants of gestational age <33 weeks between December 1998-July 2003, and found an incidence of 5.5% (five babies) in 114 babies, all presenting at various stages.^3^ Another study assessed the presence of ROP and potential risk factors other than supplementary oxygen in premature infants ≤34 wks and or ≤1500g, and detected ROP in 40% of cases (23 out of 58).^4^ Before 2010, screening for ROP was almost non-existent. Between 2010 and 2012, basic services were available for screening and laser treatment in only two centres, namely the Ispahani Islamia Eye Institute and Hospital (IIEI&H), and National Institute of Ophthalmology (NIO) in Dhaka. Only 55 babies were examined during this period at IIEI&H, of which 30% required laser treatment. Seven babies at presentation had stage V ROP, with total retinal detachment, which carries an extremely poor visual prognosis. In 2013, ORBIS International in collaboration with IIEI&H organised a stakeholders' awareness and sensitisation programme for neonatologists and other personnel. This support from ORBIS included human resources development, infrastructure and equipment. The goal of the collaboration was to ensure that no premature baby would be left out of the screening net in the NICUs of Dhaka within the next five years.

**Figure:**
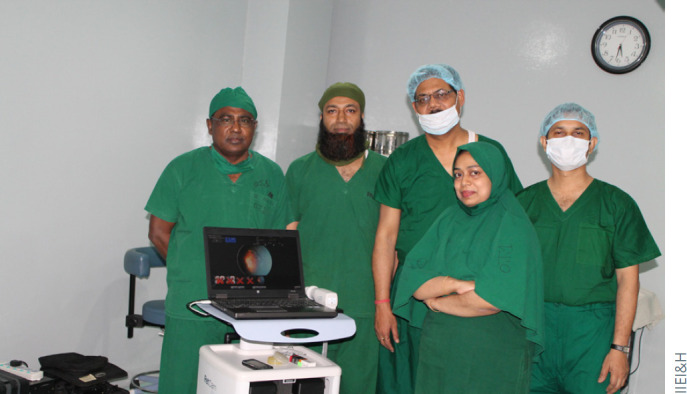
IIEI&H ROP team with Retcam. BANGLADESH

Through this programme, three retina specialists were trained in ROP at the LV Prasad Eye Institute (LVPEI) in India. ORBIS also supported the training of a ROP programme manager and technicians. The ROP programme manager is responsible for communication between the NICUs and ROP team of IIEI&H. According to a protocol, the ROP team visits NICUs to screen and treat babies before discharge. This collaboration has led to an increase in the number of babies screened. It has also reduced the proportion of babies with blinding ROP at presentation.

Currently, there are 20 NICUs in Dhaka and only three are government owned. In a survey carried out by ORBIS in 2014 at 12 NICUs of Dhaka, 2962 preterm infants were managed over a six-month period in 2014. IIEI&H screens ROP babies who are referred to the hospital from the NICU centers, and additionally performs on-site screening at three NICUs. At IIEI&H, babies with gestational age of less than 35 weeks and/or birth weight (BW) of less than 2000g are screened. Babies outside these criteria adjudged to be at risk of ROP by the neonatologist are also screened.

## Results

**Figure 1:**
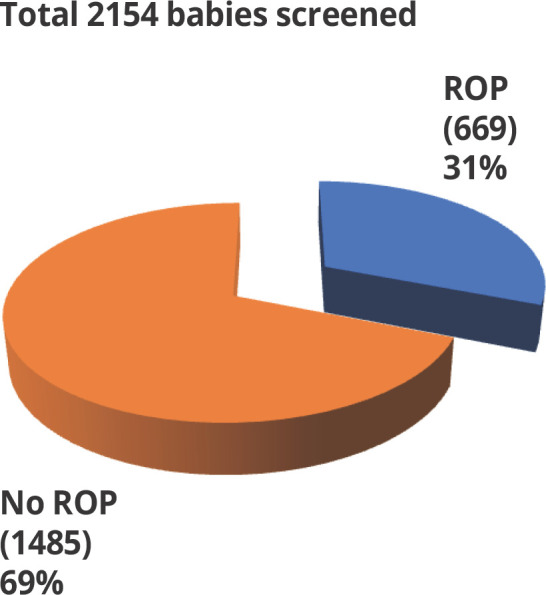
Total babies screened.

Between January 2013 and March 2017 staff in IIEIH screened over 2000 preterm infants. 40% of the babies had birth weight (BW) between 1500–2000g and 38% had BW < 1500g (Figure 1).

**Figure 2:**
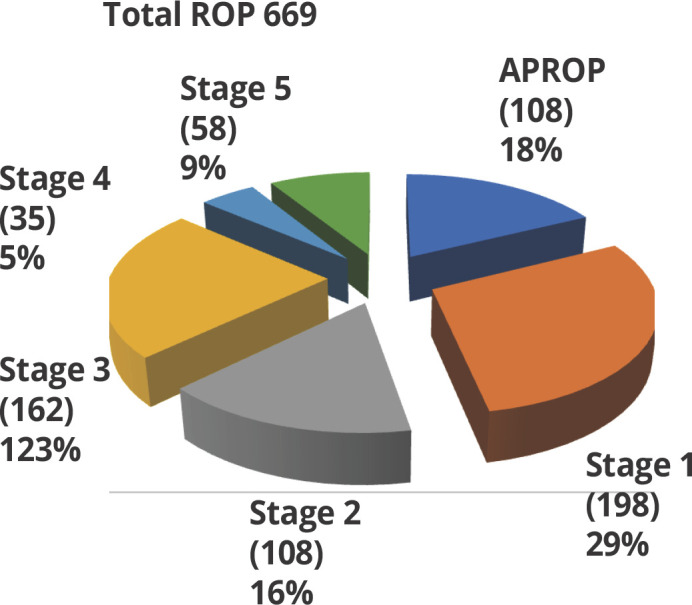
Distribution of ROP.

About a third of these babies had different stages of ROP. Stages 1 and 2 constituted 45% of the ROP cases, stage 3 was 23%, stage 4 was 5% and stage 5 was 9%. Aggressive posterior ROP occurred in 18% of all ROP cases seen at this center (Figure 2). During this period, 274 babies have been treated with laser, 69 with intraocular bevacizumab injection and 53 babies have had ROP surgery for stage 4 or 5 ROP.

The mean gestational age of babies with ROP was 31.09 ± 2.28 weeks (range: 26–36 weeks) and mean birth weight was 1354.13 ± 266.38g (range: 700–1900g). Even though our screening protocol is for babies to be screened between 20 and 30 days after birth, the mean chronological age at screening is unfortunately still very far from ideal (mean 46.63 ± 25.37 days, range: 20–150 days).

## Existing challenges

The current staff in Bangladesh includes three retinal surgeons who are proficient in ROP surgery, and 16 ophthalmologists who can diagnose and treat ROP. Four other ophthalmologists who can only screen for ROP are in districts outside Dhaka.

Five centers have equipment for laser treatment and two centers offer ROP surgical services. All these centers are located in Dhaka and are either private or NGO driven. The only RetCam in Bangladesh is in IIEI&H.

Lack of adequate trained human resources, infrastructure and equipment are major challenges. Level of awareness is still low, especially outside Dhaka. A greater level of participation from the government, better coordination between the existing centers and increased awareness of the condition, especially about the appropriate time of referral are the need of the hour.

## Future strategies

ROP training is now incorporated into retina and paediatric ophthalmology long-term fellowship programmes at IIEI&H. Short term training in ROP management is also available for ophthalmologists. We hope to develop a national ROP screening guideline in conjunction with relevant stakeholders. A national database on ROP for monitoring and evidence-based policy which will be owned by the government for continuity is imperative. We look forward to government participation in the area of infrastructure and equipment provision. Current stakeholders (both private and non-government) should aid future government programmes in light of current achievements and failures in management of ROP. There should be adequate incentives for all levels of personnel involved to sustain morale and encourage specialists at the district level.

## Conclusion

ROP is rapidly attaining public health significance in Bangladesh. There is a significant gap between the increasing need and the limited resources. The current efforts are mainly driven by the private and NGO sectors. More government involvement and commitment is required for a nationwide sustainable programme.
